# The London Medical Exhibition

**Published:** 1911-10-07

**Authors:** 


### THE LONDON MEDICAL EXHIBITION.

### POINTS THE INSTITUTIONAL OFFICER SHOULD NOTE.

The seventh London Medical Exhibition opened on Monday last at the Royal Horticultural Hall, and was attended during the week by several thousands of medical men. The profession is not slow in appreciating the valuable opportunity which such an exhibition affords of seeing for itself the advances made and the novelties brought out by the best firms dealing in surgical appliances, drugs, foods, books, and other specialities. The usual features of this now well-organised exhibition were again in evidence, but this year the promoters, *The British and Colonial Druggist*, have succeeded in gathering an even larger number of exhibitors into the Hall, of which every foot of space has been taken up eagerly.

One of the interesting exhibits is the epidiascope of Messrs. Leitz, of which frequent demonstrations were daily given. The clearness and beauty of the projections on the screen of all kinds of objects in their natural colours excited the admiration of the visitors who were wise enough to attend. Messrs. Burroughs and Wellcome have a good display of their products, including the comparatively new pituitary extract in sterilised vaporoles, epinine, lodal, tyramine, etc. Horlick's Malted Milk is now firmly established in favour with the profession and needs no introduction, though a visit to this firm's stand will impress the visitor with the wholesomeness and purity of this food. In the annexe on the right-hand side of the band is the exhibit of the Hospitals and General Contracts Co., Ltd., where a special display of several thousand instruments is to be seen, as well as coats, overalls, couches, dressings, and hospital furniture of high quality. The Medical Supply Association, of Gray's Inn Road, are showing their improved sling pillow, a contrivance as useful as it is simple. At this stand may be inspected the Grevillite Vitrenamel hospital furniture, which is made of steel and covered with an enamel which does not chip and which readily withstands the action of heat.

Virol is now used at over a thousand hospitals and institutions, which is solid testimony, and every information is given at the Virol stand concerning its preparation. Another food of quite a different nature has made rapid headway. We refer to the pure desiccated milk known as Glaxo, which, being sterilised, obviates any risk of tuberculosis. It is pure milk without any addition save cream and sugar of milk, and it is possible to rear infants upon it without their developing rickets. The reputation of Callard and Co. for diabetic foods is maintained by their display at this exhibition, which is an object-lesson in the great progress that has been made towards eliminating the reproach of dreadful monotony from the diet of diabetic sufferers. The Angier Chemical Co., Ltd., has a display of its emulsion of petroleum with hypophosphites of lime, the advantages of which over cod-liver oil continue to be recognised.

We should like to draw special attention to a new hygienic syphon made by Messrs. John Bell and Croyden, which appears to possess original merit. This syphon is of simple construction, and hygienic in a sense of that word which cannot, unfortunately, be applied to the ordinary metal-top syphon. By simply replacing a cap with a porcelain spout (retained by the purchaser) the appliance is ready for use. As the syphon is filled like an ordinary bottle there is no impediment to sterilisation; as to the efficacy of the syphon for supplying well-aërated water, practical demonstration left no doubt.

The preparations of the Aylesbury Dairy Co. are of great interest in these days, when the feeding of infants is becoming a fine study. Besides their three humanised milks, the firm displays a Lactor cream cheese and a Koumiss. The Welford Dairy Company is also exhibiting, and continues to make a speciality, as they have done for nearly a century, of asses' milk; their "Sauermilch" is prepared as recommended by Metchnikoff, with the Massol bacillus and the Streptococcus lacticus. The dangers of unclean milk are perhaps greater than those of tuberculous milk, and any sincere efforts to put on the market safe, pure, and clean milk deserve professional encouragement. The firm of B. Davies and Son, of Portman Square, makes substantial claims, and has an instructive exhibit showing the great care and supervision exercised in its dairy and laboratory.

Messrs. Stephen Smith and Co. have a large show of their Hall's Wine and Carvino, both of which preparations seem to grow in favour on account of their invigorating effects. Wincarnis is another recognised preparation, and it can now be obtained with quinine or iron or pepsin, as may be required

Of interest to hospital workers must be the preparations shown by Messrs. Keen, Robinson and Co. (incorporated with J. and J. Colman). Their patent barley makes a capital barley-water for diluting milk, and has found its way into hundreds of hospitals.

Messrs. Savory and Moore, Ltd., have had their food before the public for so long that comment is superfluous. In particular their valveless feeder deserves more than a passing mention; nurses especially will appreciate the advantages of a feeder so easily kept in order and from which the flow of milk can be regulated to a nicety.

The makers of Ivelcon and the St. Ivel lactic cheese have an exhibit that is worth seeing; the rapid success of their preparations is a reward for the care taken in the management of their model factory. There are probably few hospitals in which Izal is not in use, and Messrs. Newton, Chambers and Co. have received numerous inquiries concerning their other preparations containing this efficient disinfectant; for example, the perles containing medical Izal oil, which has proved of value in typhoid fever and cholera.

Wright's Coal Tar specialities form an imposing exhibit by themselves, but Messrs. Wright, Layman and Umney also present a fine display of aqueous tinctures and concentrated liquors which have been proved economical and satisfactory in hospital dispensaries now that there is an increased duty on spirit.

Of interest to general practitioners should be a monthly journal of therapeutics and pharmacology known as *The Prescriber*. Attention is drawn to the special offer whereby a year's subscription (5s.) and a copy of the new British Pharmaceutical Codex may be had for an inclusive sum of 10s. 6d.

The stand that is never passed by is that whereon the books of the Oxford Medical Publishers (Henry Frowde, Hodder and Stoughton) are displayed. Here one can be sure of finding some of the most important new publications and the standard text-books of all branches of medicine and surgery. Here one finds relief in scientific literature from the overwhelming mass of products the most important of which have been outlined above.

